# Optimization of the treatment with beta-lactam antibiotics in critically ill patients—guidelines from the French Society of Pharmacology and Therapeutics (Société Française de Pharmacologie et Thérapeutique—SFPT) and the French Society of Anaesthesia and Intensive Care Medicine (Société Française d’Anesthésie et Réanimation—SFAR)

**DOI:** 10.1186/s13054-019-2378-9

**Published:** 2019-03-29

**Authors:** Romain Guilhaumou, Sihem Benaboud, Youssef Bennis, Claire Dahyot-Fizelier, Eric Dailly, Peggy Gandia, Sylvain Goutelle, Sandrine Lefeuvre, Nicolas Mongardon, Claire Roger, Julien Scala-Bertola, Florian Lemaitre, Marc Garnier

**Affiliations:** 1 AP-HM Hôpital de la Timone, Service de Pharmacologie Clinique et Pharmacovigilance, 264 rue Saint Pierre, 13005 Marseille, France; 2 AP-HP Hôpital Cochin, Service de Pharmacologie, 27 rue du Faubourg St-Jacques, 75679 Paris Cedex 14, France; 3 CHU d’Amiens Picardie, Service de Pharmacologie Clinique, UPJV EA7517, Avenue Laennec, 80054 Amiens Cedex 1, France; 4 CHU de Poitiers, Département d’Anesthésie Réanimation, 2 Rue de la Milétrie, 86021 Poitiers, France; 5 CHU de Nantes, Département de Pharmacologie Clinique, 5 allée de l’île gloriette, 44093 Nantes Cedex 01, France; 6 CHU de Toulouse, Laboratoire de Pharmacocinétique et Toxicologie Clinique, Institut Fédératif de Biologie, 330, avenue de Grande-Bretagne, 31059 Toulouse cedex 9, France; 7 CHU de Lyon, Service de Pharmacie, Groupement Hospitalier Nord, Hôpital Pierre Garraud, 136 rue du Commandant Charcot, 69322 Lyon cedex 05, France; 8 CHR d’Orléans, Laboratoire de Biochimie, 14 Avenue de l’Hôpital, 45067 Orléans, France; 9 AP-HP Hôpital Henri Mondor, Département d’Anesthésie-Réanimation, 51 Avenue du Maréchal de Lattre de Tassigny, 94000 Créteil, France; 10 CHU de Nîmes, Département d’anesthésie, réanimation, douleur et médicine d’urgence, Place du Pr Robert Debré, 30029 Nîmes cedex 9, France; 11 CHRU de Nancy, Département de pharmacologie clinique et de toxicologie, 29 rue Lionnois, 54000 Nancy, France; 12 CHU Pontchaillou, Service de Pharmacologie Clinique et épidémiologique, 2 Rue Henri le Guilloux, 35000 Rennes, France; 13 AP-HP Hôpital Tenon, Département d’Anesthésie et Réanimation, 4 rue de la Chine, 75020 Paris, France

**Keywords:** Beta-lactam antibiotics, Pharmacokinetics, Pharmacodynamics, Continuous infusion, Dosage, Therapeutic drug monitoring

## Abstract

**Background:**

Beta-lactam antibiotics (βLA) are the most commonly used antibiotics in the intensive care unit (ICU). ICU patients present many pathophysiological features that cause pharmacokinetic (PK) and pharmacodynamic (PD) specificities, leading to the risk of underdosage. The French Society of Pharmacology and Therapeutics (SFPT) and the French Society of Anaesthesia and Intensive Care Medicine (SFAR) have joined forces to provide guidelines on the optimization of beta-lactam treatment in ICU patients.

**Methods:**

A consensus committee of 18 experts from the two societies had the mission of producing these guidelines. The entire process was conducted independently of any industry funding. A list of questions formulated according to the PICO model (Population, Intervention, Comparison, and Outcomes) was drawn-up by the experts. Then, two bibliographic experts analysed the literature published since January 2000 using predefined keywords according to PRISMA recommendations. The quality of the data identified from the literature was assessed using the GRADE® methodology. Due to the lack of powerful studies having used mortality as main judgement criteria, it was decided, before drafting the recommendations, to formulate only “optional” recommendations.

**Results:**

After two rounds of rating and one amendment, a strong agreement was reached by the SFPT-SFAR guideline panel for 21 optional recommendations and a recapitulative algorithm for care covering four areas: (i) pharmacokinetic variability, (ii) PK-PD relationship, (iii) administration modalities, and (iv) therapeutic drug monitoring (TDM). The most important recommendations regarding βLA administration in ICU patients concerned (i) the consideration of the many sources of PK variability in this population; (ii) the definition of free plasma concentration between four and eight times the Minimal Inhibitory Concentration (MIC) of the causative bacteria for 100% of the dosing interval as PK-PD target to maximize bacteriological and clinical responses; (iii) the use of continuous or prolonged administration of βLA in the most severe patients, in case of high MIC bacteria and in case of lower respiratory tract infection to improve clinical cure; and (iv) the use of TDM to improve PK-PD target achievement.

**Conclusions:**

The experts strongly suggest the use of personalized dosing, continuous or prolonged infusion and therapeutic drug monitoring when administering βLA in critically ill patients.

**Electronic supplementary material:**

The online version of this article (10.1186/s13054-019-2378-9) contains supplementary material, which is available to authorized users.

## Background

The use of antibiotics in the critical care setting is very common. A large multicenter European study reported antibiotic use in 64% of patients during their stay in the intensive care unit (ICU) [1]. In France, nearly half of ICU patients are treated with antibiotics on a given day [2]. ICU, together with infectious disease wards, are the two largest prescribers of antibiotics in the hospital (> 1500 defined daily doses delivered/1000 days of hospitalization) [3]. Beta-lactams are the most commonly used antibiotic class in critical care worldwide, accounting for instance for 69% of antibiotics consumed in France in 2015 [3].

The severity of infections and the frequent resistance in ICU-acquired infections, as well as the numerous pathophysiological specificities related to the critical care setting, make the conduct of beta-lactam treatment in critical care patients challenging. However, sepsis and septic shock require the rapid administration of the appropriate antibiotic at the appropriate dose. Indeed, numerous studies have shown that a delay in the administration of an appropriate antibacterial therapy is associated with increased mortality in the most severe patients [4–6]. The risk of treatment failure appears therefore to be at the same time higher and more serious than for non-critically ill patients. In this context, the unpredictability of critical care patients’ exposure to beta-lactam antibiotics for a given dose should be particularly taking into account, advocating for therapeutic drug monitoring (TDM) of beta-lactams. Available studies support this strategy, even if the clinical impact on patient’s prognosis is not yet fully demonstrated [7]. In addition, concentration targets are not consensually defined although it is an essential prerequisite for conducting further studies. However, pending large randomised clinical trials, TDM performed since the first few hours of treatment and frequently controlled on the following days may allow for a best achievement of concentration targets while limiting the risk of adverse events. Thus, in the era of personalized medicine, the individualization of beta-lactam dosage and administration regimen seems, more than elsewhere, to be required in critically ill patients. Although evidence of the beneficial clinical impact of this approach is growing in the scientific literature [8], there is currently no positioning of learned societies on this subject.

## Guidelines goals

The objective of these guidelines is to produce a framework enabling an easier decision-making process for the prescribing and monitoring of beta-lactam treatment for intensivists. The group worked to produce a minimum number of recommendations to highlight the key points to focus on the four predefined fields. In case of doubt, published data prevailed over expert opinion. The basic rules of general good medical practice were considered as known and excluded from the scope of these guidelines recommendations. The target audience corresponds to all medical professionals working in intensive care units and pharmacology laboratories.

## Methods

### General organisation

These guidelines are the result of the work provided by a panel of experts convened together by the French Society of Pharmacology and Therapeutics (Société Française de Pharmacologie et Thérapeutique; SFPT) and the French Society of Anaesthesia and Intensive Care Medicine (Société Française d’Anesthésie et Réanimation; SFAR). Each expert was required to complete a conflict-of-interest disclosure prior to participation in establishing the guidelines. The schedule of the group was defined upstream. First, the organisation committee and the guideline coordinators defined the questions to be addressed by the panelists. It then appointed experts in charge of each question. The questions were formulated according to the Patients, Intervention, Comparison and Outcome (PICO) format. The population covered by these guidelines (the PICO “P”) is the critical care patient population for all recommendations; therefore, the mention “in critically ill patients” has not been explicitly repeated in the wording of each recommendation. The literature analysis was then conducted according to the Grade of Recommendation, Assessment, Development and Evaluation (GRADE) methodology. A level-of-evidence was defined for each reference cited according to the type of study. This level-of-evidence could be re-evaluated taking into account the methodological quality of the study. Due to the small number of high-powered studies that focused on mortality, it was decided, prior to establishing these guidelines, to formulate all recommendations as “optional” recommendations (“we suggest…” or “we suggest not…”). Recommendation proposals were presented to the entire panel and discussed one-by-one. The goal was not necessarily to obtain consensus on all the proposals, but to identify points of agreement and disagreement or indecision. Each recommendation was then evaluated by each expert rated using a scale ranging from 1 (complete disagreement) to 9 (complete agreement). Collective rating was established according to a GRADE grid methodology. Only recommendations with strong agreement were considered in these guidelines, meaning that at least 70% of experts had concordant opinion, while less than 20% of them had discordant opinion. In the absence of strong agreement, recommendations were reformulated and resubmitted to reach a consensus.

## Areas of guidelines

The stated mission was to produce guidelines in four pecific areas related to the optimization of treatment with beta-lactam antibiotics in critical care patients: pharmacokinetic (PK) variability, pharmacokinetic (PK)–pharmacodynamic (PD) relationship, administration modalities, and therapeutic drug monitoring (TDM).

An extensive literature research over the period January 2000 to January 2018 was conducted by two experts for each area based on publications indexed in PubMed™, Tripdatabase (www.tripdatabase.com), PROSPERO (www.crd.york.ac.uk/PROSPERO), and clinicaltrials.gov databases. Literature review was performed using the PRISMA methodology for systematic reviews (keywords used for the bibliographic search are available in the Additional file 1). According to the GRADE methodology, a preliminary classification of outcomes has been made before reviewing the evidence using a 1–9 numerical scale, in which outcomes rated from 1 to 3 were considered as “low importance outcomes,” from 4 to 6 as “important but not critical outcomes,” and from 7 to 9 as “critical outcomes” [9]. Consequently, the judgement criteria used in our literature review were rated as follows:

- Main criteria: mortality (importance 9) and achievement of the PK-PD objective (importance 5);
- Secondary criteria: clinical cure (importance 8), microbiological cure (importance 6), mechanical ventilation free days (importance 6), length of ICU stay (importance 7), and incidence of adverse side effects (importance 7).

To be considered for analysis, publications had to be written in English or in French. The analysis was performed according to decreasing hierarchical prioritization of data from meta-analyses of randomised controlled trials (RCTs) or individual RCTs to observational studies. Study sample size and the relevance of the research were considered at the level of each study.

## Guidelines

### *First area*. Pharmacokinetic variability of beta-lactam antibiotics

Which elements of PK variability of beta-lactam antibiotics should be taken into account to decrease morbidity and mortality in ICU patients?

**R1.1. We suggest considering systematically and daily the many sources of pharmacokinetic variability when prescribing beta-lactam antibiotics to critical care patients.**

*Optional recommendation*—*strong agreement*

The use of antibiotics in critical care patients is complex as a result of the large variability of pharmacokinetic (PK) parameters and various sources of this variability.

The systemic inflammatory response syndrome (SIRS) associated with sepsis, the use of catecholamines and intravenous (IV) fluids, the types of lesions (burns, mediastinitis, etc.), the existence of organ failures (shock, renal dysfunction, liver dysfunction, etc.), and the use of extra-corporal therapies (mechanical ventilation, renal replacement therapy, extracorporeal membrane oxygenation, etc.) can substantially change the pharmacokinetics of antibiotics in ICU patients [10, 11]. The DALI multicenter international study has well illustrated the problem of PK variability of beta-lactams in the intensive care setting [12]. Considerable variability of amoxicillin, ampicillin, cefazolin, cefepime, ceftriaxone, doripenem, meropenem, and piperacillin concentrations measured in the plasma from 361 critically ill patients was observed, with concentrations that could vary by a factor of 100 from one patient to another. This study also reported the influence of this variability on the clinical response, as low plasma beta-lactam concentration was associated with reduced probability of positive clinical outcome (defined as the “completion of treatment course without change or addition of antibiotic therapy, and with no additional antibiotics commenced with 48 h of cessation”) [12]. In addition to this significant variability of PK parameters observed for the same beta-lactam antibiotic among different patients (inter-individual variability), significant variability of PK parameters for the same patient over time (intra-individual variability) has also been reported. For example, Zander et al. reported a median intra-individual variability of trough piperacillin concentrations of 30% (range, 6 to 129%) in critically ill patients after only 4 days of treatment [13]. This wide PK heterogeneity in critically ill patients induces considerable variability in antibiotic concentration for a same dose administered. This supports the need for an individualization of the beta-lactam dose in critically ill patients [14, 15].

Modifications of the volume of distribution (Vd) and renal clearance of beta-lactams are major sources of PK variability observed in critical care patients. For instance, mean Vd and clearance of cefepime could vary in ICU patients from 0.08 to 0.55 L/kg and 0.062 to 0.131 L/kg/h, respectively [16–24]. Patients’ diseases also influence antibiotic PK. For instance, Isla et al. reported higher Vd and clearance of meropenem in polytraumatised patients than in septic patients (Vd 69.5 vs. 15.7 L and clearance 54 vs. 8 L/h) [25]. Such an important variability of Vd and clearance has been reported for almost all beta-lactam antibiotics [12, 26].

Another issue is the increasingly frequent management of obese patients in the ICU. Indeed, obesity has been associated with modifications of beta-lactam PK, notably due to an increased Vd [27, 28]. Indeed, increases in adipose and lean masses and increase in blood volume contribute to increase the Vd of both lipophilic and hydrophilic (such as beta-lactams) antimicrobials [29]. Beta-lactam protein binding may also be modified in obese patients due to increased plasma concentrations of fatty acids and α1-acid glycoprotein [28]. Lastly, obese patients may present an augmented renal clearance due to the increased kidney size and renal blood flow associated with obesity [29]. Because drug dosing in obese patients is a specific issue and is not restricted to beta-lactam antibiotics and critically ill patients, formulating a specific recommendation for obese patients is out of the scope of the present guidelines and will not be further discussed.

Finally, one should remind that patient’s clinical condition may change rapidly during his ICU stay, towards either improvement and cure or degradation and organ failures. As a consequence, drug pharmacokinetics frequently change during the treatment period for a given patient, as already reported for aminoglycosides [30, 31] and beta-lactams [13].

**R1.2.1. We suggest determining the glomerular filtration rate by calculating creatinine clearance with the formula U × V/P at the onset of treatment with beta-lactam antibiotics, and every time the clinical condition and/or renal function of the patient significantly changes.**

**R1.2.2. We suggest determining the glomerular filtration rate by calculating creatinine clearance with the formula U × V/P every time beta-lactam concentration is measured in order to help in interpreting the result.**

*Optional recommendation*—*strong agreement*

Relatively few studies have conducted a rigorous analysis of the covariates influencing beta-lactam PK variability in critical care patients. Patient’s renal function and parameters set during renal replacement therapy are the most reported covariates impacting the clearance of beta-lactam antibiotics.

The SIRS presented by septic ICU patients is often accompanied by increases in cardiac and renal blood flows and in glomerular filtration rate (GFR) in the absence of acute kidney failure [32]. The administration of IV fluids and vasoactive drugs also contribute to GFR increase. All these factors contribute to augmented renal clearance (ARC) that reduces the elimination half-life of drugs excreted by the kidneys. ARC, defined as a creatinine clearance (CL_CR_) > 130 mL/min/1.73m^2^, can affect up to 40% of septic ICU patients [33–35]. Beta-lactams are hydrophilic antibiotics whose elimination is primarily renal. The increase of their renal clearance generally leads to reduce plasma concentrations [36–38]. Consequently, several algorithms for dosage adaptation of various beta-lactam antibiotics with respect to patient’s CL_CR_ have been proposed, but with incomplete effectiveness in achieving PK objectives [39, 40]. In addition, low serum albumin concentration is frequently observed in ICU patients, leading to an increase in the free fraction of the beta-lactams highly bound to plasma proteins, such as cefazoline, ceftriaxone, or ertapenem. Thus, hypoalbuminemia may lead to increased Vd and tissue penetration, and also increased elimination, of beta-lactam antibiotics by glomerular filtration and/or metabolic clearance [41]. This has been particularly observed for ceftriaxone or ertapenem [41–44].

On the opposite, renal and total plasma clearance of beta-lactams may be substantially reduced in case of acute kidney injury (AKI) [45, 46]. Although the risk of patient’s over-exposure to beta-lactams cannot be ruled out (and should be monitored), it has been suggested that this situation could actually compensate for the other factors contributing to antibiotic underexposure frequently observed in ICU patients and thus increases the probability of achieving beta-lactam concentration targets [26].

To estimate the GFR, recent French guidelines recommend the calculation of creatinine clearance using the following formula: *U*_creat_ × *V*/*P*_creat_, “*U*_creat_” being the urinary creatinine concentration (in mmol/L) measured in an urine sample collected over a period of at least 1 h, “*V*” the urinary volume expressed in mL per time unit, and “*P*_creat_” the serum creatinine concentration (in mmol/L) [32]. Indeed, estimated creatinine clearance formulas (sMDRD, CKD-EPI, Cockroft and Gault) were developed for stable patients with chronic renal insufficiency and must not be used in critically ill patients in whom normal creatininemia despite altered GFR is frequent [47–49].

**R1.3.1. We suggest measuring albumin (or at least plasma proteins) at least once at the onset of treatment with beta-lactam antibiotics in order to guide the prescription.**

**R1.3.2. We suggest measuring albumin (or at least plasma proteins) when performing beta-lactam TDM in order to help in interpreting the result.**

*Optional recommendation*—*strong agreement*

The binding of beta-lactams to albumin and plasma proteins determines the free fraction, which is the biologically active fraction that diffuses across biological membranes to tissues. The free fraction is also the fraction that is eliminated by renal and liver clearance. When plasma protein amount decreases, the capacity of beta-lactams to bind to protein decreases and beta-lactam-free fraction increases. Previous studies have shown that the binding of beta-lactams to plasma proteins in ICU patients is highly variable and is more altered for antibiotics highly bound to plasma proteins in conditions of homeostasis (e.g., ceftriaxone, cefazolin, or ertapenem) [42, 50, 51]. As a result, plasma concentration of beta-lactam antibiotics may be lowered and more unpredictable in patients with severe hypoalbuminemia. In addition, co-administration of other drugs highly bound to plasma proteins (such as sedative drugs for example) can modify protein binding of beta-lactams. Finally, conformational changes of albumin have been described in ICU patients and may result in an increased or decreased binding to proteins [52].

However, the relationship between proteins and plasma-free concentration is not straightforward. Firstly, a correlation between the free fraction and albuminemia has been shown for several beta-lactams such as flucloxacillin but is not proven for all beta-lactams. Secondly, although the increase of its free fraction increases beta-lactam antibiotic clearance, it also increases its activity and this change may have little clinical consequence if unbound concentration remains almost unchanged [53].

As a result, total and free plasma concentration of beta-lactam antibiotics is unpredictable and measuring albumin (or at least plasma proteins) could provide valuable information on the expected pharmacokinetics variability. In addition, as most laboratories currently measure the total beta-lactam concentration, protein and/or albumin level is important to measure at the same time as beta-lactam concentration in order to interpret properly TDM results and decide whether the daily dose of beta-lactam requires adaptation, especially when an intra-patient concentration variability is observed.

### *Second area.* Pharmacokinetic-pharmacodynamic relationship of beta-lactam antibiotics

Which PK-PD objectives of beta-lactam antibiotics should be targeted to decrease morbidity and mortality in ICU patients?

**R2.1. We suggest considering the percentage of the dosing interval during which the free plasma concentration of beta-lactams is above a multiple (“k”) of the minimum inhibitory concentration (MIC) of the causative bacteria (%fT > k× MIC) as the therapeutic target for treatment with beta-lactam antibiotics.**

*Optional recommendation*—*strong agreement*

Many studies performed in various animal models of infection reported that the percentage of dosing interval during which the plasma beta-lactam concentration remains above a multiple of the minimum inhibitory concentration (MIC) of the causative bacteria is the PK/PD parameter that best correlates with bactericidal activity in vivo [54, 55]. As only the free fraction of beta-lactams can diffuse into tissues, the PK-PD target is usually expressed as a percentage of dosing interval during which the free plasma concentration is above a multiple of the MIC (%*f*T > *k*× MIC).

The MIC, determined by the microbiology laboratory, is the PD reference parameter for estimating the PK-PD relationship of beta-lactam antibiotics. When the MIC of the isolated strain is not available, the use of a critical epidemiological MIC covering all the MICs of wild-type strains is recommended. In Europe, this is ECOFF (“Eucast Epidemiological Cut-OFF”) corresponding to the highest MIC for organisms devoid of phenotypically detectable acquired resistance mechanisms. ECOFF defines the upper end of the wild-type MIC distribution and should be distinguished from clinical breakpoints defining the “sensitive”, “intermediate,” or “resistant” nature of a strain, which take into account ECOFF but also the PK parameters achievable in patients and PD objectives in a standard clinical setting. Thus, using ECOFF as MIC value is more appropriate in ICU than the use of clinical breakpoints for defining PK-PD targets of a treatment with beta-lactam antibiotics. However, this reference MIC value, selected in the absence of actual MIC, can be modulated taking into account local ecology.

**R2.2. We suggest targeting a free plasma beta-lactam concentration between four and eight times the MIC of the causative bacteria for 100% of the dosing interval (fT ≥ 4–8 x MIC = 100%) to maximize bacteriological and clinical response in critical care patients.**

*Optional recommendation*—*strong agreement*

Despite experimental studies generally reported a bactericidal effect of beta-lactams for a minimum value of %*f*T > MIC between 50% and 70%, clinical data focusing on ICU patients reported favourable clinical course for higher PK-PD targets. In a large multicenter study including eight beta-lactam antibiotics, a 100% *f*T > MIC was associated with improved clinical outcome in septic ICU patients compared to 50% *f*T > MIC (OR 1.56–95%CI [1.15–2.13] vs. 1.02 [1.01–1.04], *p* < 0.03) [12]. Several other PK studies, using prospectively collected data from phase 3 randomised controlled trials, confirmed that the value of 100% *f*T > MIC was associated with improved bacteriological and clinical cure in ICU patients treated with cefepime or ceftazidime [56, 57]. In patients with infections caused by *Escherichia coli* and *Klebsiella* species treated with Cefepime, a classification and regression tree (CART) analysis showed that a *f*Cmin/MIC ratio above 7.6 was associated with bacterial eradication in 100% of patients [58]. Conversely, only 33% of the strains were eradicated when *f*Cmin/MIC was below 7.6. Other authors also reported that clinical cure in ICU patients required beta-lactam plasma concentration reaching four to six times the MIC [59, 60]. These clinical data are supported by in vitro and in vivo experimental data showing both a maximal bactericidal effect and the prevention of selection of bacterial subpopulations resistant to beta-lactams for concentrations between four and eight times the MIC [56, 57, 61–64].

Thus, a 100% *f*T ≥ MIC target seems the target minimal to achieve clinical efficacy in ICU, but a PK-PD target of *f*Cmin at least higher than four times the MIC (i.e., 100% *f*T ≥ 4× MIC) would be necessary to optimize clinical efficacy while preventing selection of resistant bacterial subpopulations. This safety margin compared to a target of 100% *f*T > MIC is also justified by (1) the inaccuracy in the determination of the MIC [65]; (2) the inaccuracy in the measurement of beta-lactam plasma concentrations of up to ± 15%; (3) the variability of the diffusion of beta-lactam antibiotics in tissues, particularly in cases of endocarditis, mediastinitis, central nervous system infections, or infections on prosthetic material; and (4) achieving the highest bactericidal rate in most cases [66].

**R2.3. For beta-lactam antibiotics without validated toxicity threshold concentration, we suggest that it is useless, and even dangerous, to exceed plasma free concentrations of beta-lactam antibiotics above eight times the MIC (i.e., %fT > 8× MIC).**

*Optional recommendation*—*strong agreement*

A strong correlation between the occurrence of seizures and the dose of beta-lactams directly injected in brain ventricles has been reported in animal models [67]. In addition, neurotoxicity of beta-lactam antibiotics has been confirmed in many case series of patients suffering from various neurological disorders such as acute confusional state, encephalopathy, myoclonus, seizures, and status epilepticus, with sometimes a fatal outcome [68, 69]. Consequently, a particular attention should be given to possible antibiotic toxicity in patients experiencing unexplained neurological manifestations, in which TDM and temporarily suspension of beta-lactam administration should be discussed.

The main risk factor associated with neurological toxicity of beta-lactam antibiotics is renal failure, which may cause rapid and significant accumulation of beta-lactams. Some molecules such as cefepime or cefazolin have a lower neurotoxicity threshold than other beta-lactam antibiotics (Table 1) [68–70]. A literature review including 37 studies representing 135 cases of neurotoxicity related to cefepime administration showed that cefepime neurotoxicity occurred in 48% of cases in patients overexposed, but in 26% of cases in patients appropriately exposed to the drug taking into account their renal function [71].

**Table 1:** Convulsing activity of beta-lactams compared to penicillin G, from [67, 69, 70]

Beta-lactam	Relative pro-convulsive activity (reference: penicillin G = 100)
Cefazolin	294
Cefepime	160
*Penicillin G*	*100*
Imipenem	71
Aztreonam	42
Ampicillin	21
Ceftazidime	17
Meropenem	16
Ceftriaxone	12
Piperacillin	11
Cefotaxime	8,8
Cefoxitine	1,8

Some studies have focused on the concentration-neurotoxicity relationship of beta-lactams in the intensive care setting. Cefepime trough concentrations above 22 mg/L (when administered by discontinuous infusions) or concentrations at steady state above 35 mg/L (when administered by continuous infusion) has been associated with neurotoxicity in 50% of patients [72, 73]. Comparatively, the same risk has been reported for trough above 64 mg/L for meropenem, 125 mg/L for flucloxacillin, and 360 mg/L for piperacillin (used without tazobactam) [74]. In combination with tazobactam, a plasma steady-state concentration of piperacillin above 157 mg/L is predictive of the occurrence of neurological disorders in ICU patients with a specificity of 97% and a sensitivity of 52% [75]. Finally, when the *f*Cmin normalized to the Eucast clinical breakpoint for *Pseudomonas* *aeruginosa* (i.e., *f*Cmin/MIC_*Pseudomonas aeruginosa*_ ratio) exceeded 8, a significant deterioration of the neurological status occurred in approximately half of the ICU patients treated with piperacillin/tazobactam and approximately two thirds of the ICU patients treated with meropenem [76]. As a result, the benefit-risk balance most likely decreases as *f*Cmin exceeds eight times the MIC.

### *Third area.* Administration of beta-lactam antibiotics

Which beta-lactam administration modalities should be used to decrease morbidity and mortality of ICU patients?

**R3.1. Pending the result of therapeutic drug monitoring (TDM), we suggest that a higher daily dose of beta-lactam antibiotics than that administered in patients outside the ICU should be administered at the onset of treatment, especially in the most critically ill patients and in those with preserved renal function.**

*Optional recommendation*—*strong agreement*

Many studies have shown that target concentrations of beta-lactam antibiotics are difficult to achieve in critical care patients using standard doses. For instance, in the study by Aubert et al., 37% of patients showed ceftazidime underexposure when administered with a dosage regimen ranging from 1 to 6 g per day depending on renal function [77]. In another study performed on 80 ICU patients in the early phase of severe sepsis and septic shock, Taccone et al. observed that PK-PD targets were achieved in only 28%, 16%, and 44% of patients treated with ceftazidime, cefepime, and piperacillin, respectively [26]. Similar results were observed for meropenem and piperacillin [78]. These studies confirmed that in ICU patients with preserved renal function, increased clearance and Vd are responsible for low beta-lactam plasma concentrations. In this context, some PK modeling studies have proposed to deliver higher beta-lactam dosing in ICU patients than the standard ones used outside the ICU. In the study by Roos et al., doses of cefepime greater than 4 g were required to achieve PK-PD targets for bacteria with high MIC such as *P. aeruginosa* [16]. Similarly, in ICU patient with ARC, high doses of ceftazidime and piperacillin up to 12 g and 24 g, respectively, were proposed [79, 80]. However, additional studies are needed to define the initial dosing regimen in this population, taking into account the factors of PK variability previously described.

Another argument for initiating treatment with high beta-lactam doses is that sepsis may be associated with modifications of beta-lactam PK at the tissue level [81]. Indeed, tissue hypoperfusion due to shock and/or vasoconstrictors may modify the tissue PK of beta-lactam antibiotics, causing an extension of the time required to reach equilibrium between plasma and tissue compartments [82, 83]. Moreover, the existence of efflux transporters that act as a barrier between the target organ and the blood, and possible tissue degradation of antibiotics has also been reported to contribute to decrease antibiotic concentration in some tissues [82].

**R3.2. We suggest administering beta-lactam antibiotics by prolonged or continuous infusions for infections due to bacteria with high MIC in order to increase the probability of achieving the PK-PD targets.**

*Optional recommendation*—*strong agreement*

Among all the studies that used the percentage of dosing interval above the MIC (% T ≥ MIC) as primary endpoint published between 2000 and 2018, five studies were conducted according to a sufficiently robust methodology to assess the relevance of continuous infusion compared to discontinuous administration in infections due to high MIC bacteria.

For four of them [84–87], the methodology was similarly subdivided into three stages: (1) creating and validating a population pharmacokinetic (POP PK) model based on plasma concentrations measured in volunteers according to the usual administration regimen; (2) from the POP PK model, 10,000 kinetic profiles were simulated using Monte Carlo simulations according to different administration regimen and dosing; and (3) then the percentage of simulated profiles reaching a defined percentage of time above the MIC (known as the “Probability of Target Attainment” or “PTA”) was calculated for an extended range of MIC values. The MIC from which the tested dosage regimen (including the dosing, frequency of administration, and continuous vs. discontinuous administration) is no longer deemed “pharmacologically efficient” is defined as the MIC for which the PTA becomes less than 90% or 95% (PTA_90%_, PTA_95%_) for a given % *f*T ≥ MIC. In Krueger’s study, continuous meropenem administration achieved the PK target of 40% *f*T ≥ MIC for bacteria with MIC ≤ 4 mg/L (at a dose of 3 g/24 h) and ≤ 2 mg/L (at a dose of 1.5 g/24 h), while intermittent administration only achieved the same target for bacteria with MIC ≤ 0.5 mg/L (3 g/24 h) and ≤ 0.25 mg/L (1.5 g/24 h) [84]. Similar results were obtained with imipenem, for which a continuous administration of 2 g/24h achieved the PK target of 40% *f*T ≥ MIC for bacteria with MIC < 4 mg/L, while intermittent administration of 1g × 3/24 h only achieved the same target for MIC <2mg/L [85]. In Landersdorfer’s study, continuous or prolonged administration over 4 h of 6 g/24h of flucoxacillin achieved the PK target of 50% *f*T ≥ MIC for bacteria with MIC < 1 mg/L, while administration of the same daily dose by 30-min infusions only achieved the same target for MIC < 0.375 mg/L [86]. In De Jongh’s study, continuous infusion of 4 g/24h of temocillin achieved the PK target of 40% *f*T ≥ MIC for bacteria with MIC ≤ 16 mg/L, while 30-min infusions of 2 g twice daily only achieved the same target for MIC ≤ 8 mg/L [87].

Finally, in a randomised study that compared continuous vs. discontinuous administration of piperacillin/tazobactam, all patients treated with continuous infusion of 13.5 g/24h had a free piperacillin concentration far above the highest MIC observed (i.e., 100% *f*T > MIC), while patients treated with discontinuous infusions of 3.375 g/6 h had free piperacillin concentration above the MIC for just 50% of the dosing interval (i.e., 50% *f*T > MIC) [88].

Although there is no consensus when it comes to defining a “high” MIC, it seems reasonable to consider that a “high” MIC for a given beta-lactam antibiotic is a MIC value above the median of the distribution of MIC values for wild-type strains of the considered bacteria. For instance, a MIC > 0.125 mg/L for cefotaxime and > 2 mg/L for piperacillin/tazobactam for *E. coli* and a MIC > 2 mg/L for ceftazidime and > 4 mg/L for piperacillin/tazobactam for *P. aeruginosa* may be considered as high MIC.

**R3.3. We suggest administering beta-lactam antibiotics by prolonged or continuous infusions in critical care patients with septic shock and/or a high severity score in order to improve the clinical cure rate.**

*Optional recommendation*—*strong agreement*

In contrast to the meta-analyses conducted prior to 2010 that have not reported any benefit of the continuous infusion [89, 90], the most recent meta-analysis conducted by Lee et al., which included 13 randomised controlled trials focusing on ICU patients suffering from respiratory infections, showed an improvement in terms of clinical cure in septic patients (RR 1.194, 95%CI [1.015–1.405]) and in patients at high risk of mortality (APACHE II score ≥ 20 or SAPS II score ≥ 52) (RR 1.162 [1.042–1.296]) treated with beta-lactam continuous infusion [91]. However, no difference was observed for the mortality rate for both septic patients and patients at high risk of mortality. Similar results were observed in the meta-analysis performed by Lal et al. in 2016 focusing on nosocomial pneumonia due to Gram-negative bacteria [92]. While no difference in mortality was observed, a significantly higher clinical cure rate was noted in ICU patients with an APACHE II score > 15 treated with beta-lactam continuous infusion compared to those treated with discontinuous infusions (OR 3.45 [1.08–11.01]). Similarly, the meta-analysis performed by Roberts et al. in 2016 focusing on ICU patients with severe sepsis showed a significant improvement of the clinical cure rate in patients with an APACHE II score ≥ 22 treated with continuous beta-lactam infusion (RR 1.40 [1.05–1.87]) [93]. Although there was no significant difference in terms of ICU mortality rates between the two methods of administration in this subgroup of most severe patients (RR 0.79 [0.53–1.17]), there was a strong trend towards reducing 30-day hospital mortality with continuous administration (RR 0.74 [0.53–1.01], *p* = 0.06). In addition, the meta-analysis conducted by Teo et al. in 2013 before the availability of the BLING II and BLISS studies, also reported for the most severe patients with an APACHE II score ≥ 15 improved clinical cure (RR 1.26 [1.06–1.50]) and mortality (RR0.63 [0.48–0.81]) [94]. Finally, a recent meta-analysis focusing on anti-pseudomonal beta-lactam antibiotics (i.e., carbapenems, piperacillin-tazobactam, cefepime, and ceftazidime) reported reduced mortality in septic patients treated with extended/continuous compared to intermittent beta-lactam administration (RR 0.70 [0.56–0.87]) [95]. This effect was particularly demonstrated for the most critically ill patients (8 studies, 977 patients with APACHE II score > 20; RR 0.73 [0.57–0.94] vs. 2 studies, 302 patients with APACHE II score < 20; RR 0.72 [0.28–1.80]) [95].

Apart from these meta-analyses, other studies have shown an improvement in ICU patient outcome associated with continuous beta-lactam administration. In the single-center, open-label, randomised study conducted by Fan et al. in ICU patients treated with piperacillin/tazobactam, overall mortality rate on day 14 was similar for patients from the “4-hour prolonged administration” and “30-minute intermittent administration” groups (11.5% vs. 15.7%, *p* = 0.29). However, in the most severe patients with an APACHE II score ≥ 29.5 and who had infectious organisms isolated, prolonged infusions of piperacillin/tazobactam was associated with lower mortality rate than intermittent infusions (12.9% vs. 40.5%, *p* = 0.01) [96]. The retrospective cohort study conducted by Winstead et al. on 181 patients also highlighted that continuous administration of piperacillin/tazobactam to treat Gram-negative bacterial infections in patients with an APACHE II score ≥ 17 was associated with significant decreased hospital mortality and 30-day re-admission rates (OR_adj_ 0.20 [0.07–0.57]) [97]. The post-hoc analysis carried out by Abdul-Aziz et al. from the DALI cohort confirmed a higher clinical cure rate (prolonged administration 73% vs. intermittent 35%, *p* = 0.035) and 30-day survival rate (73% vs. 25%, *p* = 0.025) in patients with a SOFA score ≥ 9 and treated with continuous infusion of piperacillin/tazobactam or meropenem [98]. In a prospective randomised study that included ICU patients with severe sepsis, continuous administration of meropenem, piperacillin/tazobactam, and ticarcillin/clavulanic acid was associated with an improved clinical cure rate when compared to intermittent beta-lactam administration (70% vs. 43%, *p* = 0.037), although there was no effect on 90-day mortality [99]. Finally, in the observational before-after study conducted by Lodise et al. comparing extended infusions over 4 h to 30-min intermittent infusions of piperacillin/tazobactam in patients suffering from *P. aeruginosa* infections, a significant decrease in mortality was observed in the subgroup of the most critically ill patients with an APACHE II score ≥ 17 treated with extended infusions (12.2% vs. 31.6%, *p* = 0.04) [100].

All these results demonstrate, at least, an improvement in the clinical cure rate with continuous administration of beta-lactam antibiotics in the most critically ill patients. However, a single-specific threshold defining a “severe patient” cannot be recommended given the disparity of the severity scores and cut-off values used in the literature.

**R3.4. We suggest administering beta-lactam antibiotics by prolonged or continuous infusions in critically ill patients suffering from lower respiratory tract infections in order to improve the clinical cure rate.**

*Optional recommendation*—*strong agreement*

Two meta-analyses highlighted a significant improvement in the clinical cure rate of ICU patients suffering from lower respiratory tract infections treated by continuous administration of beta-lactam antibiotics compared to intermittent administration (RR 1.177 [1.065–1.300]—patients with lower respiratory tract infections [91]; and OR 2.45; [1.12–5.37]—nosocomial pneumonia due to Gram-negative bacteria [92]), although there was no effect on mortality.

The prospective randomised BLISS study showed in the subgroup of patients with severe sepsis due to pneumonia an improvement in the clinical cure rate (59% vs. 33%, *p* = 0.022) and more ventilator-free days at day 28 (22 [0–24] vs. 14 [0–24], *p* = 0.043) for patients treated with continuous infusion vs. intermittent bolus [101]. The prospective randomised, open-label study led by Fan et al. showed a significant decrease in mortality on day 14 in patients with pneumonia and treated by 4-h prolonged infusions of piperacillin/tazobactam compared to 30-min intermittent boluses (8.9% vs. 18.7%, *p* = 0.02) [96]. Finally, the post-hoc analysis performed by Abdul-Aziz et al. in the DALI cohort found a greater 30-day survival rate in patients with lower respiratory tract infections treated with prolonged beta-lactam administration compared to intermittent administration (86% vs. 57%, *p* = 0.012) [98].

**R3.5. We suggest administering beta-lactam antibiotics by prolonged or continuous infusions in critically ill patients suffering from infections due to non-fermenting Gram-negative bacilli in order to improve the clinical cure rate.**

*Optional recommendation*—*strong agreement*

In the meta-analysis led by Roberts et al. based on the individual data from 632 ICU patients suffering from severe sepsis included in three randomised controlled trials (BLING I, BLING II and BLISS), more than two thirds of the infections were due to Gram-negative bacteria (GNB) [93]. In this meta-analysis, there was a clear correlation between infections due to non-fermenting GNB and 30-day hospital mortality (OR 2.72 [1.32–5.62], *p* = 0.01), while infection due to non-fermenting GNB was an independent covariate included in the final Cox regression model demonstrating an improved survival rate with continuous compared to intermittent beta-lactam administrations. Similarly, a negative correlation between the presence of GNB and (i) clinical cure rate (*p* = 0.036) and (ii) 30-day survival (*p* = 0.039) was also found in the DALI study [98]. The superiority of continuous beta-lactam administration is also reported in the meta-analyses conducted by Lee et al. [91] and Lal et al. [92] in which respiratory infections were mainly due to GNB. In addition, three retrospective studies included in the meta-analysis by Lal et al. [92] or included in the systematic review added by Lee et al. at the end of their meta-analysis [91] also showed an improved clinical cure rate in patients with infections due to GNB with the highest MIC treated with continuous beta-lactam administration [102–104].

Focusing more specifically on *P. aeruginosa*, several studies reported concordant trends suggesting that prolonged or continuous beta-lactam administration may be beneficial for the treatment of *P. aeruginosa-*related infections in ICU patients. Indeed, in Fan et al. study 14-day mortality for the subgroup of patients infected with *P. aeruginosa* was 10% for prolonged vs. 26% for intermittent piperacillin/tazobactam infusions, respectively (*p* = 0.17) [96]. In the BLISS study, Abdul-Aziz et al. found a better clinical cure rate of *P. aeruginosa*-related infections treated with continuous beta-lactam administration [52% vs. 25% for 30-min intermittent administration (*p* = 0.052)] [101]. Finally, the same trend was noted in the study performed by Lodise et al. with a mortality rate of 8.8% in patients treated with prolonged administration vs. 15.2% in patients treated with intermittent boluses (*p* = 0.17), this difference becoming significant in the most severe patients with an APACHE II score ≥ 17 (12.2% vs. 31.6%, *p* = 0.04) [100].

**R3.6. We suggest administering an intravenous loading dose before starting the continuous or prolonged infusion at the onset of treatment with beta-lactam antibiotics, in order to achieve a concentration within the PK-PD targets as quickly as possible.**

*Optional recommendation*—*strong agreement*

**Fig. 1:**
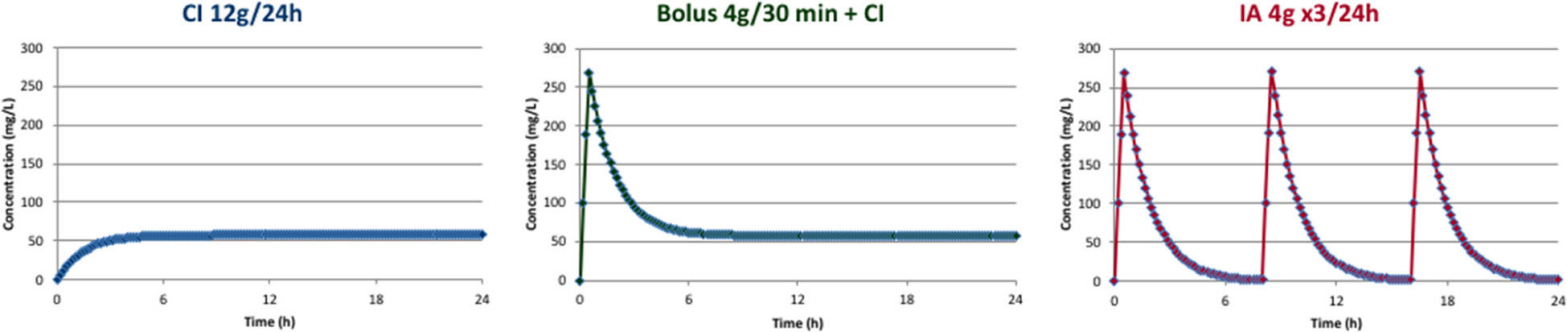
Simulated plasma concentrations obtained for a 12 g piperacillin daily dose delivered as intermittent administrations (IA, right) or continuous infusion without (CI, left) or with a loading dose (Bolus+CI, middle). The continuous infusion preceded by a loading dose is the regimen that achieves the greatest % *f*T ≥ MIC. The trough concentration before the next injection falls below the MIC in the discontinuous administration regimen, while the concentration may remain below the MIC for several hours after the beginning of the infusion in the case of continuous administration without a loading dose.

Simulations of kinetic profiles using population pharmacokinetic models according to different administration regimens demonstrate the interest of the administration of a beta-lactam loading dose before the initiation of a continuous infusion, in order to reach both an expected steady-state concentration and a “pharmacologically effective” concentration as quickly as possible (cf. Fig. 1).

In addition, nearly all the clinical and/or pharmacokinetic studies investigating continuous beta-lactam administration mentioned the use of a loading dose prior to continuous infusion (at least 25 publications from 2000 to 2018 including the most recent BLISS and BLING II studies, cf. *GRADE table summarizing the evidence provided as* Additional file 1). Conversely, this was not the case in the study conducted by Kollef et al. comparing a fixed 7-day course of doripenem 1 g as a 4-h infusion every 8 h with a fixed 10-day course of imipenem-cilastatin 1 g as a 1-h infusion every 8 h to treat ventilator-associated pneumonia (VAP) due to GNB [105]. The study was stopped prematurely due to lower clinical efficacy and increased mortality at day 28 in the doripenem arm, especially for VAP due to *P. aeruginosa*. Without being able to formally conclude on the imputability of the absence of a loading dose in these results, the longer delay necessary to achieve an effective carbapenem concentration in the absence of loading dose may have played a role [105, 106]. Finally, in the meta-analysis conducted by Vardakas et al., a subgroup analysis showed a significant reduction in mortality only when a loading dose was administered before starting the continuous infusion (13 studies with loading dose, RR 0.63 [0.47–0.84] vs. four studies without loading dose, RR 0.56 [0.17–1.85]) [95].

Although there is currently no consensus on the dose of beta-lactam antibiotic to be used for the loading dose, it seems reasonable and pragmatic to propose the administration of a loading dose identical to that used in the case of discontinuous administration, followed by immediate start of the continuous infusion.

In addition, we suggest that the use of a loading dose should be independent of the continuous or intermittent administration of beta-lactam antibiotics. Indeed, the antibiotic concentration measured just after the loading dose depends on its volume of distribution that is in great majority increased in critically ill patients, in particular in the most severe with multiple organ failures and highly increased capillary permeability. Thus, using a loading dose for the first beta-lactam administration should be encouraged in all critically ill patients, independently of the presence of organ failure or of the continuous or discontinuous administration of the antibiotic. Then, the total daily dose should be discussed according to the presence of organ failure, and notably of acute kidney injury, as this is the main organ conditioning beta-lactam clearance and consequently the steady-state concentration. These data also support our recommendation to adjust the daily dose to the clearance of creatinine, while always using a loading dose corresponding to a unitary dose as used during discontinuous administration in a patient without kidney failure.

#### Addenda to the third area

(1) Regarding the safety profile, clinical studies have not shown any significant difference in terms of frequency (e.g., diarrhoea, skin rash, phlebitis) or severity (e.g., level of renal failure) of adverse reactions attributable to beta-lactam antibiotics administered by either continuous/prolonged or discontinuous infusions.

(2) Although continuous or prolonged infusion of beta-lactam antibiotics may be preferable in the above-mentioned indications, continuous administration must take into account the chemical stability of these antibiotics over time [107]. This is of particular importance for carbapenems that have a stability of a few hours (imipenem/cilastatin: 2–3 h, ertapenem and meropenem: from 6 to 12 h depending on the reconstitution concentration) at 25°C in 0.9% NaCl [107, 108], requiring administration of the daily dose in several divided doses prepared just before their infusion.

### *Fourth area.* Therapeutic drug monitoring of beta-lactam antibiotics

Which modalities of therapeutic drug monitoring of the treatment with beta-lactam antibiotics should be used to decrease morbidity and mortality of ICU patients?

**R4.1. We suggest performing therapeutic drug monitoring in ICU patients with expected beta-lactam PK variability and/or in patients with clinical signs potentially related to beta-lactams toxicity.**

*Optional recommendation*—*strong agreement*

Few studies have assessed the impact of beta-lactam therapeutic drug monitoring (TDM) in ICU patients, and none of these studies reported an impact on clinical outcome [109–111]. However, drug exposure and PK-PD target attainment have been shown to be higher when using TDM. Due to the large PK variability of beta-lactam antibiotics reported in ICU patients (cf. *first area*), TDM appears then as an important tool to avoid drug under- or overdosage, as recently described by Wong et al. [112].

**R4.2. We suggest performing therapeutic drug monitoring (TDM) of beta-lactam antibiotics in critical care patients undergoing renal replacement therapy.**

*Optional recommendation*—*strong agreement*

The incidence of AKI in ICU patients is about 40%, requiring renal replacement therapy (RRT) in about one patient out of five [113]. RRT can cause considerable changes in antibiotic PK [114].

There are three main techniques of RRT: continuous veno-venous hemodialysis (CVVHD) (based on the principle of diffusion through a semi-permeable membrane driven by a concentration gradient), continuous veno-venous hemofiltration (CVVH) (based on the principle of convection through a filtration membrane), and continuous veno-venous hemodiafiltration (CVVHDF) (based on the combination of the two previous techniques). The type of technique and flow rates used has a direct impact on the elimination of dialyzable beta-lactams. However, antibiotic elimination rate for a given technique and flow rate changes depending on the molecule. For instance, Valtonen et al. showed that the half-lives of piperacillin and tazobactam were significantly shorter with CVVHDF compared to CVVH (6.1 ± 2 h vs. 7.7 ± 2.3 h for piperacillin; 9.4 ± 2.4 h vs. 13.9 ± 3.9 h for tazobactam) [115]. Consequently, the fraction of the dose eliminated after 12 h of RRT was higher for CVVHDF than for CVVH. Furthermore, elimination of piperacillin and tazobactam was markedly impacted by the dialysis flow-rate set during CVVHDF. By comparison, mean total plasma clearance of piperacillin during CVVHDF was 5.5 ± 2.1 L/h, representing about 50% of the clearance observed in healthy volunteers [116].

RRT is therefore a particularly challenging condition for optimal beta-lactam dosing. In addition to the variability of beta-lactam concentration due to the extent of extraction of the antibiotic, the physicochemical properties of the molecule and its interactions with the filtration membrane are also involved. Furthermore, antibiotic binding to plasma protein also impacts beta-lactam elimination rate because only the free fraction diffuses though filtration membranes. In this context, although the effect of hypoalbuminemia on beta-lactam PK in patients with preserved renal function has been documented [41], little information exists on its impact in patients undergoing RRT. Only one study has investigated the free concentrations of ertapenem and showed increased clearance of the drug in the case of hypoalbuminemia, but without any significant pharmacodynamic impact [117]. Finally, residual renal function of patients requiring RRT is variable and difficult to assess. It is rarely considered when performing TDM, despite its potential contribution to the clearance of beta-lactam antibiotics. Indeed, the involvement of a residual renal elimination during RRT has been described for piperacillin [46, 118], meropenem [117], and doripenem [119]. For instance, the total clearance of piperacillin was increased fivefold in patients with residual CL_CR_ > 50 mL/min compared with patients with residual CL_CR_ < 10 mL/min [46].

All these findings make it very difficult to propose general recommendations for beta-lactam dosing in patients undergoing RRT, even for each technique separately. Thus, personalized TDM appears necessary.

**R4.3. We suggest performing beta-lactam TDM by dosing plasma trough concentration in case of intermittent administration and plasma steady-state concentration in case of continuous administration.**

**R4.4. We suggest performing beta-lactam TDM 24 to 48 h after the onset of treatment; after any change in dosage; and in the event of a significant change in the patient’s clinical condition.**

*Optional recommendation*—*strong agreement*

To assess the percentage of the dosing interval during which the free plasma concentration of beta-lactams is above a multiple of the MIC, it is suggested to measure plasma trough concentrations in case of intermittent administration and plasma steady-state concentrations in case of continuous administration. Steady state is reached after five times the half-life, corresponding globally to 24 to 48 h after the onset of any beta-lactams antibiotic treatment and before 24 h when a loading dose is used.

Any significant modification of circulatory, renal, or hepatic functions; inflammatory conditions; and therapeutic interventions (such as fluid expansion, albumin or catecholamine administration, and kidney replacement therapy) may change beta-lactam antibiotics PK and should lead to repeat TDM.

**R4.5. In case of central nervous system infection, we suggest performing beta-lactam TDM, if possible, on blood and cerebrospinal fluid samples collected concomitantly.**

*Optional recommendation*—*strong agreement*

In case of central nervous system infection (meningitis, encephalitis, cerebral abscess, ventricular drain infection), the preferred sample is cerebrospinal fluid (CSF) in order to assess drug diffusion into the brain, with a target beta-lactam concentration in the CSF above the MIC of the isolated bacteria. The large majority of beta-lactam antibiotics have limited diffusion through the blood-brain barrier [120]. Although diffusion is increased in case of meningeal inflammation, CSF beta-lactam concentrations are hardly predictable. Indeed, a wide variability of the CSF-to-blood concentration ratio has been reported in ICU patients treated for central nervous system infection [121, 122]. Thus, individual beta-lactam TDM in the CSF performed 24 to 48 h after the onset of treatment may be very useful to ensure adequate antibiotic exposure.

It should be noted that TDM in the CSF is not an indication to perform a dedicated lumbar puncture. CSF sampling specifically dedicated to beta-lactam TDM may be considered in patients with external ventricular drain. In addition, in the event that a control lumbar puncture is indicated due to poor clinical evolution, unusual involved bacteria such as *Staphylococcus* or *Pseudomonas*, or involved bacteria with high MIC, a part of the CSF sample should be dedicated to perform beta-lactam TDM. In the future, the potential use of cerebral microdialysis could be a novel method for monitoring regional brain concentrations of beta-lactam antibiotics [123].

**R4.6.1. We suggest performing beta-lactam TDM according to a validated chromatographic method.**

**R4.6.2. We suggest that beta-lactam TDM results should be available to clinicians as soon as possible in order to have a real impact on ICU patient’s management.**

*Optional recommendation*—*strong agreement*

A chromatographic method validated according to the European Medical Agency guidelines [124] with a mass spectrometry or a diode-array detection should be preferred for measuring plasma beta-lactam concentration [125, 126].

TDM results should be available as soon as possible, allowing rapid adjustment of the beta-lactam dosage in case of under- or over-exposure. Currently, in many laboratories, the results of beta-lactam plasma concentration monitoring are mostly available only after several days, which is of retrospective interest only and does not have a significant impact on the patient’s clinical course. Ideally, having results available within 24 h of sampling would be an objective to be pursued. Indeed, recent analytical methods allow a rapid determination of beta-lactam concentrations and would help to reach the objectives of an early TDM actually belonging to daily care [127]. Further, the potential use of a closed loop system in the near future may offer a new way for real-time antibiotic concentration monitoring [128].

**R4.7. We suggest considering as therapeutic targets the plasma concentrations presented in** **Table** **2***.*

**Table 2:** Target trough total (Cmin) or free (*f*Cmin) plasma concentration following intermittent administration and target total (Css) or free (*f*Css) steady-state plasma concentration following continuous administration for the main beta-lactam antibiotics

	Free fraction (%)	Recommended target concentrations^#^		MIC threshold^£^ [130]	Ref.
		Documented infection	Non-documented infection		
Amoxicillin	≈ 80%	*f*Cmin or *f*Css ≥ 4× MIC Cmin or Css < 80 mg/L	Cmin 40–80^*^mg/L^§^ Css 40–80 mg/L	8 mg/L (ECOFF *E. coli*)	[131]
Cefazolin	≈ 15–20%	*f*Cmin or *f*Css ≥ 4× MIC Cmin or Css < 80 mg/L	Cmin 40–80 mg/L^§^ Css 40–80 mg/L	2 mg/L (ECOFF *S. aureus*)	[132]
Cefepime	80%	*f*Cmin or *f*Css ≥ 4× MIC Cmin < 20 mg/L Css < 35 mg/L	Cmin 5–20 mg/L Css 5–35 mg/L	1 mg/L (*Enterobacteriaceae*)^§§^	[21, 72, 73]
Cefotaxime	≈ 60–80%	*f*Cmin or *f*Css ≥ 4× MIC Cmin or Css < 60 mg/L	Cmin 25–60 mg/L Css 25–60 mg/L	4 mg/L (ECOFF *S. aureus*)	[133]
Ceftazidime	≈ 90%	*f*Cmin or *f*Css ≥ 4× MIC Cmin or Css < 80 mg/L	Cmin 35–80 mg/L^§^ Css 35–80 mg/L	8 mg/L (ECOFF *P. aeruginosa*)	[77]
Ceftriaxone	≈ 10%	*f*Cmin ≥ 4× MIC Cmin < 100 mg/L	Cmin 20–100 mg/L	0.5 mg/L (ECOFF *E. cloacae*)	[129]
Cloxacillin	≈ 10%	*f*Cmin or *f*Css ≥ 4× MIC Cmin ou Css < 50 mg/L	Cmin 20–50 mg/L^§^ Css 20–50 mg/L	0.5 mg/L (ECOFF *S. aureus*)	[131]
Ertapenem	≈ 10%	*f*Cmin ou *f*Css ≥ 4× MIC Cmin < 10 mg/L	Cmin 5–10 mg/L	0.125 mg/L (*H. influenzae*)^§§§^	[117, 134]
Imipenem	≈ 80%	*f*Cmin ≥ 4× MIC Cmin < 5 mg/L	Cmin 2.5–5 mg/L	0.5 mg/L (ECOFF *E. coli*)	[135]
Meropenem	≈ 100%	*f*Cmin ou *f*Css ≥ 4× MIC Cmin ou Css < 16 mg/L	Cmin 8–16 mg/L^§^ Css 8–16 mg/L	2 mg/L (ECOFF *P. aeruginosa*)	[136]
Piperacillin	≈ 80%	*f*Cmin ou *f*Css ≥ 4× MIC Css < 160 mg/L	Css 80–160 mg/L	16 mg/L (ECOFF *P. aeruginosa*)	[75]

£The Minimum Inhibitory Concentration (MIC) threshold was chosen by considering the treatment with beta-lactam antibiotics either (i) during the empirical phase or (ii) in the case of no microbiological documentation, when the beta-lactam antibiotic administered is the object of a clinical bet to cover a maximum of the bacterial species usually identified in the considered infection

**#**The highest values of the targets should be considered for infections of tissues in which beta-lactam diffusion is reduced (endocarditis, infection of prosthetic material, mediastinitis, etc.)

*The target trough free plasma concentration of four to eight times the MIC is 32 to 64 mg/L considering a MIC threshold set at 8 mg/L (*E. coli* Epidemiological Cut-OFF (ECOFF) for amoxicillin). As the free fraction is about 80% of the total dose, the target trough total plasma concentration is estimated at 40 to 80 mg/L. *The same calculation has been made for all the other beta-lactam antibiotics taking into account their binding to plasma proteins and the considered MIC threshold*

**§**In this situation, the minimal target trough plasma concentration is difficult to achieve by intermittent administration, encouraging to prefer a continuous administration in order to reach this target

**§§** The highest ECOFF value (8 mg/L for *P. aeruginosa*) was not considered to calculate the target plasma concentration, since this would have resulted in a concentration above the clinically defined toxic threshold. To be consistent with the maximal plasma concentrations that could be achieved without neurological toxic effect [21, 72, 73], the clinical breakpoint for *Enterobacteriaceae* (1 mg/L, which is the higher ECOFF value except for *P. aeruginosa*) was considered to estimate the target

**§§§** The highest ECOFF value (1 mg/L for *S. aureus*) was not considered to calculate the target plasma concentration, since this would have resulted in a concentration not consistent with the plasma concentrations usually reported [117, 134]. To be consistent with the plasma concentrations usually reported in the literature, the clinical breakpoint for *H. influenzae* (0.125 mg/L, which is the higher ECOFF value except for *S. aureus*) was considered to estimate the target

*Optional recommendation—strong agreement*

We previously suggested targeting a free plasma beta-lactam concentration between four and eight times the MIC of the involved bacteria for 100% of the dosing interval (*f*T ≥ 4–8× MIC = 100%) in order to maximize bacteriological and clinical response in critical care patients (Cf. *R2.2*). In documented infections with determination of the MIC for the identified bacterium, the available MIC should be used. In documented infection without available MIC, the ECOFF value for the identified bacterium should be considered. During the empirical phase of the treatment with beta-lactam antibiotics (i.e., when the bacteriological documentation is not yet available) or if the infection is not documented (i.e., in the absence of microbiological sampling or in the case of inconclusive sampling), the highest ECOFF value among those of the bacteria usually involved in the considered infection should be used.

To limit the toxicity of beta-lactam antibiotics, the maximum target beta-lactam concentrations have been determined from previously published relationships between concentrations and toxicity, and toxicity thresholds for trough or steady-state concentrations. Such data are available for cefepime [72, 73], ceftriaxone [129], and piperacillin [75]. For the other molecules, for which such data are not available, a trough free plasma concentration equal to eight times the MIC was proposed as the upper value of the target. Indeed, higher beta-lactam concentrations did not improve efficacy [63] and increased the risk of neurologic toxicity [76] (Cf. *R2.4*).

**R4.8.1. In case of non-achievement of the target beta-lactam plasma concentration, we suggest in first line:**

**- Either increasing the frequency of administration (i.e., further fractionate the dose) or switching to continuous administration, while maintaining the same daily dose;**

**- Or increasing the unit dose administered discontinuously by 25 to 50% while maintaining the same frequency of administration.**

**R4.8.2. In the case of persistence of below-target beta-lactam plasma concentration despite one of the previous measures, we suggest switching to prolonged or continuous administration in combination with an increase of the beta-lactam daily dose.**

*Optional recommendation*—*strong agreement*

Below are the common arguments with R4.9.

**R4.9.1. In case of supra-therapeutic plasma beta-lactam concentration, we suggest in first line:**

- **Either reducing the daily dose in the case of continuous administration;**
- **Or decreasing the unit dose administered discontinuously by 25 to 50% while maintaining the same frequency of administration.**

**R4.9.2. In case of extremely high concentration and/or signs of toxicity consistent with beta-lactam overdose, we suggest stopping the administration and further resuming the treatment after having checked the decrease in beta-lactam concentration; then conducted under strict TDM.**

**R4.9.3. We suggest performing renal replacement therapy if acute renal failure is, at least partially, responsible for symptomatic beta-lactam overdose.**

*Optional recommendation*—*strong agreement*

In the case of underdosage and due to the time-dependent pharmacokinetics of beta-lactam antibiotics, it makes pharmacologically sense to increase in first line: (i) the frequency of administration (or even to switch to continuous infusion) of the same daily dose or (ii) the unit dose with the same frequency of administration (thus increasing the daily dose administered). In case of persistent underdosage, the two previous recommendations should be combined by increasing the daily dose to be administered continuously.

For the same reason, it makes pharmacologically sense to prefer the reduction of the administered unit dose, while maintaining the frequency of administration in the case of beta-lactam overdose without significant accumulation and/or toxic signs. In the event of significant accumulation and/or clinical signs of toxicity related to beta-lactam overdose, the administration should be immediately stopped. The treatment can be resumed at lower dose only after having controlled that plasma beta-lactam concentration has decreased within the therapeutic range. If at least a part of beta-lactam overdose can be attributed to acute renal failure, initiating renal replacement therapy should be considered in order to reduce the elimination half-life and hasten the elimination of dialysable beta-lactam antibiotics.

Good practice rules imply that all suspected cases of beta-lactam toxicity should be declared to the pharmacovigilance center.

## Conclusions

**Fig. 2:**
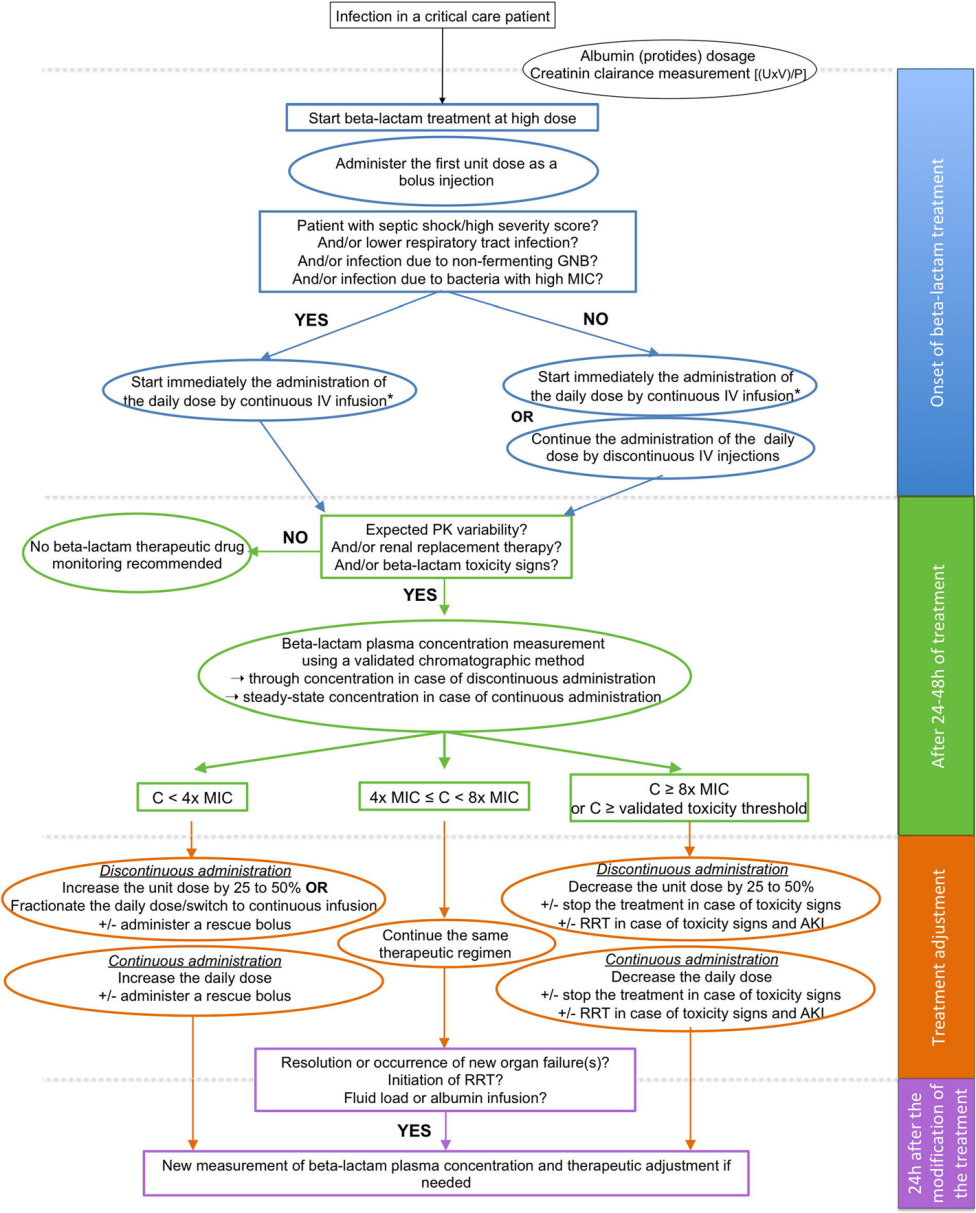
Care protocol suggested by the experts (*expert opinion*, *strong agreement*). *Taking into account the stability of the administered beta-lactam after its reconstitution, i.e., using several syringes continuously infused IV per day

The expert panel analyses and application of the GRADE method led to 21 optional recommendations and one care protocol (Fig. 2), all gathering a strong agreement, highlighting the need for personalized medicine when administering beta-lactam antibiotics in the ICU setting. The most important messages regarding beta-lactam administration in critically ill patients concerned (i) the consideration of the many sources of PK variability in this population; (ii) the definition of free plasma concentration between four and eight times the MIC of the causative bacteria for 100% of the dosing interval as PK-PD target to maximize bacteriological and clinical responses; (iii) the use of continuous or prolonged administration of beta-lactam antibiotic in the most severe patients, in case of high MIC bacteria and in case of lower respiratory tract infection to improve clinical cure; and (iv) the use of TDM to improve PK-PD target achievement.

## Organizers and expert coordinators

SFPT: Romain Guilhaumou, Service de Pharmacologie Clinique et Pharmacovigilance Hôpital de la Timone, AP–HM, 13005 Marseille, France.

SFAR: Marc Garnier, Département d’anesthésie et réanimation, Hôpital Tenon, AP-HP, 75020 Paris, France.

## Experts panel

SFPT: Romain Guilhaumou (Marseille), Florian Lemaitre (Rennes), Sihem Benaboud (Paris), Eric Dailly (Nantes), Sylvain Goutelle (Lyon), Sandrine Lefeuvre (Orléans), Youssef Bennis (Amiens), Peggy Gandia (Toulouse), Julien Scala Bertola (Nancy), Guillaume Deslandes (Nantes), Ronan Bellouard (Nantes), Matthieu Grégoire (Nantes), Clément Boidin (Lyon), Parastou Moshiri (Orléans), Sandra Bodeau (Amiens).

SFAR: Marc Garnier (Paris), Claire Dahyot-Fizelier (Poitiers), Claire Roger (Nîmes), Nicolas Mongardon (Créteil).

## Review panels

SFPT Therapeutic Drug Monitoring and Treatment Personalisation Group: Romain Guilhaumou, Stéphane Bouchet, Damien Montange, Fabien Lamoureux, Elodie Gautier.

SFPT Scientific Committee: Véronique Leblais, Patrick Rossignol, Caroline Victorri-Vigneau, Régis Bordet, Laurence Daulhac Terrail, Françoise Stanke-Labesque, Pierre-Olivier Girodet, Agnès Sommet, Alain Carriou.

SFAR Guidelines Committee: Lionel Velly, Marc Garnier, Julien Amour, Alice Blet, Gérald Chanques, Vincent Compère, Philippe Cuvillon, Fabien Espitalier, Etienne Gayat, Hervé Quintard, Bertrand Rozec, Emmanuel Weiss.

SFAR Intensive Care Committee: Marc Leone, Sébastien Mirek, Yazine Mahjoub, Antoine Virat, Antoine Roquilly, Laurent Muller, Matthieu Legrand, Caroline Duracher Gout, Christophe Quesnel, Olivier Joannes Boyau, Arnaud Friggeri, Ségolène Mrozek, Claire Dahyot-Fizelier, Olivier Langeron, Jean-Michel Constantin, Jean-Christophe Orban.

Guidelines reviewed and endorsed by the SFPT (04/07/2018) and SFAR (21/06/2018) boards.

## Additional files

Additional file 1: GRADE Table - First area - PK variability provides the GRADE Table summarizing the methods and the results of the studies taken into consideration to formulate the recommendations of the first area of the guidelines: “Pharmacokinetic variability of beta-lactam antibiotics”. GRADE Table - Second area – PK-PD provides the GRADE Table summarizing the methods and the results of the studies taken into consideration to formulate the recommendations of the second area of the guidelines: “Pharmacokinetic-Pharmacodynamic relationship of beta-lactam antibiotics”. GRADE Table - Third area – Administration of beta-lactams provides the GRADE Table summarizing the methods and the results of the studies taken into consideration to formulate the recommendations of the third area of the guidelines: “Administration of beta-lactam antibiotics”. GRADE Table - Fourth area – TDM provides the GRADE Table summarizing the methods and the results of the studies taken into consideration to formulate the recommendations of the fourth area of the guidelines: “Therapeutic Drug Monitoring of beta-lactam antibiotics”. Flow-charts of study seletion provides a flow chart of the selection of the relevant studies among all the studies identified by the literature search for each of the four areas of the guidelines. Supplementary method file provides the keywords used for the bibliographic search for each of the four areas of the guidelines. (ZIP 134 kb)
