# Supplementary material for: Optimization of the treatment with beta-lactam antibiotics in critically ill patients—guidelines from the French Society of Pharmacology and Therapeutics (Société Française de Pharmacologie et Thérapeutique—SFPT) and the French Society of Anaesthesia and Intensive Care Medicine (Société Française d’Anesthésie et Réanimation—SFAR)
Source: Crit Care. 2019 Mar 29;23:104. doi: 10.1186/s13054-019-2378-9 (PMC6441232; doi:10.1186/s13054-019-2378-9)
Supplement: Supplementary file 1 — GRADE Table - First area - PK variability provides the GRADE Table summarizing the methods and the results of the studies taken into consideration to formulate the recommendations of the first area of the guidelines: “Pharmacokinetic variability of beta-lactam antibiotics”. GRADE Table - Second area – PK-PD provides the GRADE Table summarizing the methods and the results of the studies taken into consideration to formulate the recommendations of the second area of the guidelines: “Pharmacokinetic-Pharmacodynamic relationship of beta-lactam antibiotics”. GRADE Table - Third area – Administration of beta-lactams provides the GRADE Table summarizing the methods and the results of the studies taken into consideration to formulate the recommendations of the third area of the guidelines: “Administration of beta-lactam antibiotics”. GRADE Table - Fourth area – TDM provides the GRADE Table summarizing the methods and the results of the studies taken into consideration to formulate the recommendations of the fourth area of the guidelines: “Therapeutic Drug Monitoring of beta-lactam antibiotics”. Flow-charts of study seletion provides a flow chart of the selection of the relevant studies among all the studies identified by the literature search for each of the four areas of the guidelines. Supplementary method file provides the keywords used for the bibliographic search for each of the four areas of the guidelines. (ZIP 134 kb) [file 13054_2019_2378_MOESM1_ESM.zip › Supplementary method file.docx]

**Optimization of the treatment with beta-lactam antibiotics in critically ill patients**

*Guidelines from the French Society of Pharmacology and Therapeutics (Société Française de Pharmacologie et Thérapeutique - SFPT) and the French Society of Anaesthesia and Intensive Care Medicine (Société Française d’Anesthésie et Réanimation - SFAR)*

**Supplementary method file**

Keywords used for the bibliographic search.

For all areas: « critically ill patient » OR « critical care » OR « ICU »

**First area**. Pharmacokinetic variability of beta-lactam antibiotics

- « Pharmacokinetics » AND «variability» AND « critically ill patient »
- « Beta lactam » AND «variability» AND « critically ill patient »
- « Beta lactam » AND « concentration » AND « critically ill patient »
- « Beta lactam » AND « pharmacokinetics » AND « critically ill patient »

**Second area**. Pharmacokinetic-Pharmacodynamic Relationship of beta-lactam antibiotics

- « Beta lactam » AND « continuous infusion »
- « Beta lactam » AND « intermittent infusion »
- « Beta lactam » AND « prolonged infusion »
- « Beta lactam » AND « loading dose »

**Third area**. Administration of beta-lactam antibiotics

- « Beta lactam » AND « Pharmacokinetic/Pharmacodynamic » AND « critically ill patient »
- « Beta lactam » AND « Pharmacokinetic/Pharmacodynamic  »
- « Beta lactam » AND « neurotoxicity » AND « critically ill patient »
- « Beta lactam » AND « toxicity » AND « critically ill patient »
- « Beta lactam » AND « adverse drug reaction » AND « critically ill patient »
- « Beta lactam » AND « efficacy » AND « critically ill patient »
- « Beta lactam » AND « outcome » AND « critically ill patient »

**Fourth area**. Therapeutic Drug Monitoring of beta-lactam antibiotics

- « Beta lactam » AND « therapeutic drug monitoring » AND « critically ill patient »
- « Beta lactam » AND « therapeutic drug monitoring »
- « Beta lactam » AND « modeling » AND « critically ill patient »
- « Beta lactam » AND « population pharmacokinetic » AND « critically ill patient »
- « Beta lactam » AND « Bayesian » AND « critically ill patient »
